# Changes in Nutrient Accumulation and Transportation of Waxy Sorghum in Waxy Sorghum-Soybean Intercropping Systems Under Different Row Ratio Configurations

**DOI:** 10.3389/fpls.2022.921860

**Published:** 2022-07-22

**Authors:** Can Wang, Lingbo Zhou, Jie Gao, Guobing Zhang, Fangli Peng, Chunlan Zhang, Qiang Zhao, Qiu Peng, Mingbo Shao

**Affiliations:** Institute of Upland Food Crops, Guizhou Academy of Agricultural Sciences, Guiyang, China

**Keywords:** intercropping, row ratio configurations, waxy sorghum, nutrient accumulation, nutrient transportation, yield

## Abstract

To determine the optimal row ratio configuration of waxy sorghum-soybean intercropping systems, a field experiment with seven treatments, including sole waxy sorghum (SW), sole soybean (SS), two rows of waxy sorghum alternated with one row of soybean (2W1S), two rows of waxy sorghum alternated with two rows of soybean (2W2S), three rows of waxy sorghum alternated with one row of soybean (3W1S), three rows of waxy sorghum alternated with two rows of soybean (3W2S), and three rows of waxy sorghum alternated with three rows of soybean (3W3S), was conducted during 2019 and 2020 in Guiyang, China. Accumulation and transportation of nitrogen (N), phosphorus (P), and potassium (K) in waxy sorghum were investigated. The results showed that the row ratio configurations had significant effects on the N, P, and K accumulation and transportation of waxy sorghum. On the one hand, compared to SW treatment, intercropping treatments showed higher N, P, and K contents and accumulation amounts, N, P, and K transportation amounts before anthesis, N, P, and K transportation rates before anthesis, and contribution rates of N, P, and K transportation before anthesis to the grain of each organ in waxy sorghum. Similarly, the waxy sorghum-soybean intercropping system increased the yield components (including spike length, grain number per spike, and 1,000-grain weight) of waxy sorghum. In addition, the yields of waxy sorghum and soybean among all treatments were in the sequence of SW (SS) > 2W1S > 3W1S > 3W2S > 3W3S > 2W2S. Besides, the 2W1S treatment showed the highest land equivalent ratio and economic benefit. On the whole, the waxy sorghum-soybean intercropping system can increase the N, P, and K absorption among organs and promote the N, P, and K transportation from vegetative organs to grain in waxy sorghum so as to promote the growth and development of spike in waxy sorghum to obtain higher land equivalent ratio and economic benefits. The 2W1S treatment was recommended as the optimal row ratio configuration of the waxy sorghum-soybean system to achieve the maximum utilization of nutrient resources.

## Introduction

Waxy sorghum [*Sorghum bicolor* (L.) Moench], a cereal grain crop, is planted mainly as a main raw material for brewing Moutai-flavor liquor in Southwest China, especially in Guizhou province (Wang et al., 2017; Zhou et al., 2020). For the past few years, with the demand for poverty alleviation and the adjustment of agricultural structure in Guizhou, the planting area of waxy sorghum is increasing year by year. However, due to the limited cultivated land in karst mountainous areas, waxy sorghum continuous cropping has become an important factor restricting the sustainable production of waxy sorghum (Fan et al., 2015, 2016). Therefore, finding a reasonable and sustainable planting pattern to break the continuous cropping obstacle is essential for the development of waxy sorghum production.

Intercropping is a method of planting two or more crops in the same field to realize the intensification of time and space (van Oort et al., 2020), which is a profitable way of producing more yields on the same piece of land by the most efficient utilization of resources in both temporal and spatial arrangements (Rusinamhodzi et al., 2012; Tetteh et al., 2019). Reasonable intercropping can not only make effective use of natural resources such as light, temperature, water, heat, and gas but also fully exploit the potential of soil and promote the absorption and utilization of nutrients by plants so as to improve the yield and quality, which is of great significance for alleviating the contradiction between increasing population and decreasing land area and promoting the sustainable development of land resources (Nelson et al., 2018; Bukovsky-Reyes et al., 2019). Row ratio configuration may be a more important agronomic measure in the management of intercropping systems because it can alter the field microclimate of intercrops, affect the competition relationship between intercropped species, and thus determine the crop yields (Tan et al., 2020). Cereal and legume intercropping is widely recognized as a sustainable agricultural production system that can increase crop yield and reduce chemical fertilizer input by promoting the symbiotic nitrogen fixation capacity of legumes (Liu et al., 2015; Gong et al., 2020). Sorghum is a gramineous and C_4_ crop with high photosynthetic capacity (Baena et al., 2017). Soybean is a C_3_ legume with high symbiotic nitrogen fixation ability, which can reduce the carbon footprint of cropping systems (Li et al., 2019b). Thus, exploring the appropriate row ratio configuration of the waxy sorghum-soybean intercropping system is an important step to improve the yield and quality of waxy sorghum.

At present, there are many reports on the intercropping planting mode of sorghum and legume crops. For example, Gebremichael et al. (2019) showed that sorghum intercropped with pigeon pea and cowpea increased the land productivity as its land equivalent ratio was >1, which indicated that legume crops contributed to the yield of sorghum either intercropped with legume or grown using the residual contribution of legumes after a year. Arshad et al. (2020) indicated that the grain yield of sweet sorghum was significantly reduced in intercropping with mungbean but remained on par between sole cropping and intercropping with soybean. Wang et al. (2021a) reported that row ratio configurations in waxy sorghum-soybean intercropping affected the land equivalent ratio by altering photosynthetically active radiation and the leaf area index to regulate leaf photosynthetic characteristics, dry matter formation, and the spike structure of waxy sorghum. Liang et al. (2021) showed that sorghum and soybean intercropping had higher land productivity, and water, and nitrogen utilization advantages. Wang et al. (2021b) indicated that sorghum intercropped with peanut and soybean promoted the accumulation of sorghum biomass and increased the comprehensive economic yield of the intercropping system. Nitrogen (N), phosphorus (P), and potassium (K) are the essential mineral nutrients for crop growth and development (Rosen et al., 2014; Chen et al., 2015; Tsialtas et al., 2016). Some researchers documented that intercropping promoted the absorption and accumulation of N, P, and K in crops compared to sole cropping (Li et al., 2013; Raza et al., 2019; Fan et al., 2020). However, no study has been carried out to understand the nutrient accumulation and transportation of waxy sorghum in the waxy sorghum-soybean intercropping systems under different row ratio configurations. Thereby, the objectives of this study were to (1) evaluate the yield performance of waxy sorghum under different row ratio configurations in the waxy sorghum-soybean intercropping system and (2) investigate the effects of different row ratio configurations on nutrient (N, P, and K) accumulations and transportations of waxy sorghum in the waxy sorghum-soybean intercropping systems.

## Materials and Methods

### Experimental Site

**Figure 1:**
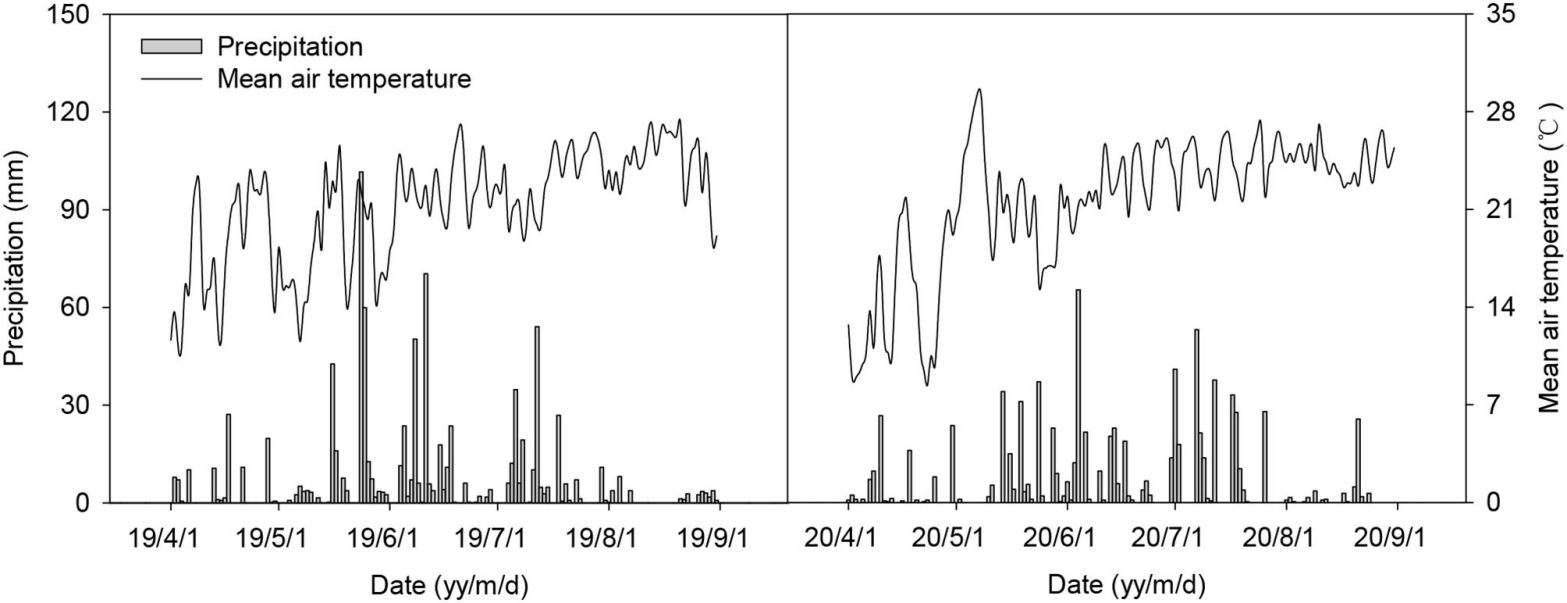
The mean air temperature and precipitation during the experiment period.

The field experiments were conducted in 2019 and 2020 at the Experimental Farm of Guizhou Academy of Agricultural Sciences (26°32′N, 106°48′E), Guiyang, China at an elevation of 1,139 m above sea level. The site has a plateau monsoon climate with an annual mean temperature of 15.6°C, annual precipitation of 1,450.8 mm, and annual sunshine duration of 1,287.4 h. The mean air temperature and precipitation during the experiment period are shown in Figure 1. The soil is sandy loam with a pH of 7.52, organic matter of 15.81 g kg^−1^, total N of 1.62 g kg^−1^, total P of 1.05 g kg^−1^, total K of 23.22 g kg^−1^, available N of 133.05 mg kg^−1^, available P of 22.14 mg kg^−1^, and available K of 400.18 mg kg^−1^ in the 0–100 mm soil layer at the start of this experiment in 2019.

### Experimental Design

The waxy sorghum cultivar “Qiangao 7” and soybean cultivar “Qiandou 7” currently used in local production were used in the experiment. Field experiments were conducted using a randomized complete block design with seven treatments and three replicates. The treatments were sole waxy sorghum (SW), sole soybean (SS), two rows of waxy sorghum alternated with one row of soybean (2W1S), two rows of waxy sorghum alternated with two rows of soybean (2W2S), three rows of waxy sorghum alternated with one row of soybean (3W1S), three rows of way sorghum alternated with two rows of soybean (3W2S), and three rows of way sorghum alternated with three rows of soybean (3W3S). For all treatments, the row spacing between waxy sorghum and soybean rows was 60 cm, the distance between adjacent waxy sorghum and soybean rows was 60 cm, the distance between two adjacent waxy sorghum holes and two adjacent soybean holes in a row was 25 cm, and the length of each experimental plot was 5 m. Both SW and SS treatments were four rows per plot with a plot size of 12 m^2^. Intercropping treatments included three strips and the plot sizes of 2W1S, 2W2S, 3W1S, 3W2S, and 3W3S were 30, 39, 42, 51, and 60 m^2^, respectively. Waxy sorghum and soybean in all treatments were sown and harvested at the same time. Sowing was performed on 8 April 2019 and 10 April 2020, and final singling was performed at the five-leaf stage to a uniform specification of two plants per hole. Harvesting was performed on 20 August 2019 and 24 August 2020. A compound fertilizer (contained 14% N, 16% P_2_O_5_, and 15% K_2_O) was used as a basal fertilizer at a dose of 450 kg ha^−1^ at sowing time, and urea (containing 46.4% N) was used as additional fertilizer at a dose of 300 kg ha^−1^ at the jointing stage of waxy sorghum. The previous crop in the first experimental year was oilseed rape and other farming measures were used according to the farmer's practices.

### Measurements of Nutrients (N, P, and K)

Six waxy sorghum plants in three holes were selected randomly from a middle strip of each plot at the jointing stage, the anthesis stage, and the maturity stage of waxy sorghum. The selected plants were uprooted with a small hole, and the roots of waxy sorghum were washed up with running tap water. Then, the root, stem (containing sheath), leaf, and spike of each sampled plant were collected and placed in an oven for 30 min at 105°C to kill the fresh tissues and then dried to a constant weight at 80°C. The dry matter weight (DW) of each sample was measured with an electronic balance (Heeyii JE-301, Hangzhou, China), and the data were reported in our previous studies (Wang et al., 2021a). Next, each dried sample was powdered using a powder machine (Kefeng FW-100, Zhengzhou, China) by passing through a 60-mesh sieve, and 0.2 g of powder sample was digested in 70% concentrated H_2_SO_4_ and 30% H_2_O_2_. The N content was measured using the Kjeldahl method (Hibbard, 1910), the P content was measured using the vanadium-molybdenum yellow colorimetry (Zhou et al., 2006), and the K content was measured using the flame photometry (Yildiz et al., 2010). The nutrient accumulation amount (NA), the nutrient transportation amount before anthesis (NTA), the nutrient transportation rate before anthesis (NTR), and the contribution rate of nutrient transportation before anthesis to grain (GCRNT) were calculated using the following formulas described by Hu et al., 2018:


(1) $$\begin{array}{c} \;\\ \;\\ \; \end{array}\begin{array}{l} NA\;=\;DW\;\times \;NC\\ NTA\;=\;NAA\;-\;NAM\\ NTR\;=\;\frac{NTA}{NAA}\;\times \;100\%\\ \begin{array}{c} GCRNT\;=\;\frac{NTA}{GNA}\;\times \;100\%, \end{array} \end{array}$$

where NC is the nutrient content of each organ, NAA is the nutrient accumulation amount of the vegetative organ at the anthesis stage, NAM is the nutrient accumulation amount of the vegetative organ at the maturity stage, and GNA is the nutrient accumulation amount of the grain at the maturity stage.

### Measurements of Yield and Economic Benefits

At the maturity stage, six waxy sorghum plants were selected randomly from a middle strip of each plot to measure yield components. Spike length was measured as the distance from the base of the spike to the top of the spike. Grain number per spike and 1,000-grain weight were measured with an automatic seed analysis system (Wanshen SC-G, Hangzhou, China). All waxy sorghum and soybean plants were hand harvested to determine yields, and the economic benefit was converted by 10 CNY kg^−1^ of waxy sorghum and 6 CNY kg^−1^ of soybean. The land equivalent ratio (LER) was calculated using the following formula:


(2) $$\begin{array}{r} LER\;=\;\frac{Y_{iw}}{Y_{sw}}\;+\;\frac{Y_{is}}{Y_{ss}}, \end{array}$$

where Y_iw_ and Y_is_ are the yields of intercropped waxy sorghum and soybean, respectively, and Y_sw_ and Y_ss_ are the yields of sole waxy sorghum and soybean, respectively.

### Data Analysis

The analysis of variance was performed with SPSS 19 software (SPSS Institute Inc., Illinois, USA), and data from each sampling data were analyzed separately. Means were tested by the least significant difference at the *P* < 0.05 level (LSD_0.05_). Figures were drawn by SigmaPlot 10 software (Aspire Software Intl., Ashburn, USA).

## Results

### N Accumulation and Transportation of Waxy Sorghum

#### N Content

The N contents of waxy sorghum vegetative organs (including root, culm, and leaf) decreased from the jointing stage to the maturity stage in all treatments (Table 1). In each treatment, the order of N content among organs was leaf > culm > root at the jointing stage, spike > leaf > culm > root at the anthesis stage, and grain > leaf > culm > root at the maturity stage, respectively. In each growth stage, the N content of each organ among treatments was in the sequence of 2W1S > 3W1S > 3W2S > 3W3S > 2W2S > SW, and the 2W1S treatment significantly increased the mean N content in 2 years by 44.49% for roots, 50.4% for culms, and 40% for leaves at the jointing stage; 94.44% for roots, 109.98% for culms, 89.44% for leaves, 80.31% for spikes at the anthesis stage; and 87.69% for roots, 64.26% for culms, 59.12% for leaves, and 58.77% for grains at the maturity stage compared with the SW treatment.

**Table 1:** Effects of different row ratio configurations on N content (g kg^−1^) of waxy sorghum in the waxy sorghum-soybean intercropping system.

**Organ**	**Treatment**	**2019**			**2020**		
		**Jointing stage**	**Anthesis stage**	**Maturity stage**	**Jointing stage**	**Anthesis stage**	**Maturity stage**
Root	SW	11.64 ± 0.43 c	4.67 ± 0.30 e	2.23 ± 0.26 d	11.31 ± 0.40 d	4.65 ± 0.35 e	2.18 ± 0.24 d
	2W1S	16.63 ± 0.46 a	9.18 ± 0.34 a	4.18 ± 0.08 a	16.52 ± 0.60 a	8.93 ± 0.40 a	4.10 ± 0.19 a
	2W2S	12.68 ± 0.98 c	6.00 ± 0.33 d	2.82 ± 0.32 cd	12.46 ± 1.26 cd	5.89 ± 0.38 d	2.69 ± 0.23 cd
	3W1S	16.21 ± 0.10 a	8.23 ± 0.56 ab	3.78 ± 0.28 ab	16.11 ± 1.19 ab	7.78 ± 0.42 b	3.59 ± 0.31 ab
	3W2S	15.23 ± 0.73 ab	7.56 ± 0.33 bc	3.68 ± 0.25 ab	15.12 ± 1.16 abc	6.96 ± 0.13 bc	3.24 ± 0.08 bc
	3W3S	13.52 ± 0.70 bc	6.91 ± 0.05 cd	3.36 ± 0.24 bc	13.39 ± 1.43 bcd	6.31 ± 0.25 cd	2.96 ± 0.10 c
Culm	SW	17.54 ± 1.71 d	4.38 ± 0.41 e	2.26 ± 0.40 e	17.42 ± 1.63 c	3.56 ± 0.17 e	2.19 ± 0.48 d
	2W1S	27.66 ± 0.77 a	9.32 ± 0.28 a	4.46 ± 0.11 a	26.27 ± 0.46 a	8.79 ± 0.52 a	4.44 ± 0.23 a
	2W2S	19.26 ± 1.49 cd	4.81 ± 0.18 de	2.67 ± 0.27 de	18.26 ± 2.98 c	4.14 ± 0.30 de	2.53 ± 0.41 cd
	3W1S	23.78 ± 1.20 b	7.84 ± 0.45 b	3.78 ± 0.15 b	23.52 ± 1.76 ab	7.25 ± 0.54 b	3.80 ± 0.39 ab
	3W2S	23.37 ± 0.76 b	6.61 ± 0.49 c	3.22 ± 0.29 c	23.02 ± 3.28 ab	5.42 ± 0.26 c	3.18 ± 0.38 bc
	3W3S	21.51 ± 0.58 bc	5.54 ± 0.28 d	3.72 ± 0.58 d	20.85 ± 2.75 bc	4.71 ± 0.22 d	2.80 ± 0.22 cd
Leaf	SW	29.11 ± 0.66 d	18.39 ± 1.94 d	9.58 ± 0.17 d	29.04 ± 0.33 e	15.87 ± 0.34 d	9.47 ± 0.99 e
	2W1S	37.70 ± 1.04 a	32.88 ± 2.32 a	13.74 ± 1.00 a	37.69 ± 0.68 a	28.48 ± 0.77 a	13.49 ± 0.17a
	2W2S	31.09 ± 1.29 cd	20.01 ± 1.25 d	10.36 ± 1.55 cd	30.53 ± 1.39 d	17.85 ± 2.46 cd	10.04 ± 0.97 de
	3W1S	36.66 ± 0.51 a	27.26 ± 1.64 b	12.76 ± 0.69 ab	36.58 ± 0.16 ab	25.22 ± 0.92 b	12.58 ± 1.01 ab
	3W2S	35.53 ± 5.25 ab	24.28 ± 2.88 c	11.97 ± 0.87 bc	35.44 ± 0.48 b	23.57 ± 2.95 b	12.05 ± 1.09 bc
	3W3S	33.36 ± 1.63 bc	22.97 ± 2.72 c	11.44 ± 0.88 bc	32.52 ± 1.17 c	20.20 ± 0.87 c	10.97 ± 0.47 cd
Spike	SW		12.83 ± 0.76 d	14.39 ± 0.61 d		12.58 ± 0.50 d	14.25 ± 1.07 d
(Grain)	2W1S		20.66 ± 1.72 a	23.17 ± 0.45 a		20.45 ± 0.51 a	22.20 ± 1.18 a
	2W2S		15.50 ± 0.58 bcd	15.21 ± 0.94 d		14.58 ± 0.42 cd	15.16 ± 0.31 d
	3W1S		18.18 ± 1.22 ab	20.30 ± 0.89 b		17.91 ± 0.21 b	19.97 ± 1.40 b
	3W2S		16.56 ± 0.13 bc	18.96 ± 0.75 bc		16.42 ± 0.59 bc	18.44 ± 1.20 c
	3W3S		15.32 ± 0.51 cd	18.35 ± 1.62 c		15.30 ± 1.12 c	17.32 ± 0.95 c

*Data are expressed as the mean of three replicates ± standard error (n = 3). Different letters within a column and an organ indicate significant differences among treatments (P < 0.05). SW, sole waxy sorghum; 2W1S, two rows of waxy sorghum alternated with one row of soybean; 2W2S, two rows of waxy sorghum alternated with two rows of soybean; 3W1S, three rows of waxy sorghum alternated with one row of soybean; 3W2S, three rows of way sorghum alternated with two rows of soybean; 3W3S, three rows of way sorghum alternated with three rows of soybean*.

#### N Accumulation

**Figure 2:**
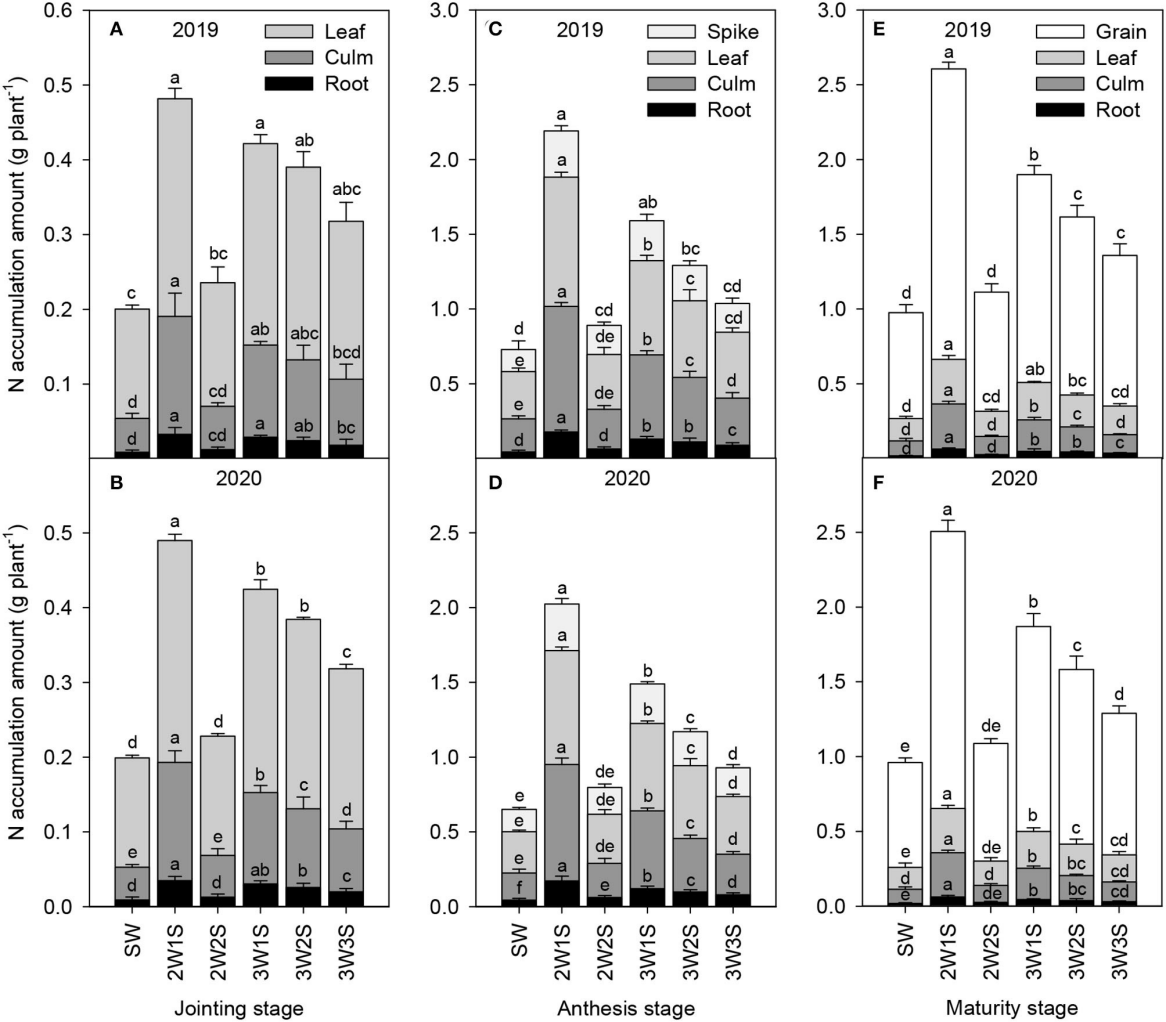
Effects of different row ratio configurations on N accumulation amount of waxy sorghum at the jointing **(A,B)**, anthesis **(C,D)**, and maturity **(E,F)** stage in the waxy sorghum-soybean intercropping system. Data are expressed as the mean of three replicates and bars represent standard errors (*n* = 3). Different letters within a growth stage and an organ indicate significantly differences among treatments (*P* < 0.05). SW, sole waxy sorghum; 2W1S, two rows of waxy sorghum alternated with one row of soybean; 2W2S, two rows of waxy sorghum alternated with two rows of soybean; 3W1S, three rows of waxy sorghum alternated with one row of soybean; 3W2S, three rows of way sorghum alternated with two rows of soybean; 3W3S, three rows of way sorghum alternated with three rows of soybean.

The N accumulation amounts of waxy sorghum vegetative organs (including root, culm, and leaf) increased from the jointing stage to the anthesis stage but decreased from the anthesis stage to the maturity stage in all treatments (Figure 2). Across years and treatments, the order of N accumulation amount among organs was leaf > culm > root at the jointing stage (Figures 2A,B), spike > leaf > culm > root at the anthesis stage (Figures 2C,D), and grain > leaf > culm > root at the maturity stage (Figures 2E,F), respectively. In each growth stage, the N accumulation amount of each organ under intercropping treatments was higher than that of SW treatment, and the maximum value appeared in the 2W1S treatment. In particular, the N accumulation amount (mean of 2 years) in the 2W1S treatment was higher by 3.83 times for roots, 3.59 times for culms, and 2.43 times for leaves at the jointing stage, 3.95 times for roots, 4.01 times for culms, 3.32 times for leaves, and 3.05 times for spikes at the anthesis stage, and 3.32 times for roots, 3.12 times for culms, 2.5 times for leaves, and 2.64 times for grains at the maturity stage than that of SW treatment.

#### N Transportation

There had been significant effects on the NTA, NTR, and GCRNT of waxy sorghum by row ratio configurations in the waxy sorghum-soybean intercropping system (Table 2). In each treatment, the NTA, NTR, and GCRNT among organs were in the sequence of leaf > culm > root, root > culm > leaf, and leaf > culm > root, respectively. In both years, the NTA, NTR, and GCRNT of each organ among treatments were in the sequence of 2W1S > 3W1S > 3W2S > 3W3S > 2W2S > SW. Compared with the SW treatment, the 2W1S treatment significantly increased the mean NTA, NTR, and GCRNT of 2 years by respectively 341.02, 12.17, and 64.95% for roots, 389.6, 24.11, and 82.02% for culms, and 249.70, 23.99, and 33.64% for leaves.

**Table 2:** Effects of different row ratio configurations on the N transportation amount before anthesis (NTA), the N transportation rate before anthesis (NTR), and the contribution rate of N transportation before anthesis to grain (GCRNT) of waxy sorghum in the waxy sorghum-soybean intercropping system.

**Organ**	**Treatment**	**NTA (mg plant^−1^)**		**NTR (%)**		**GCRNT (%)**	
		**2019**	**2020**	**2019**	**2020**	**2019**	**2020**
Root	SW	25.77 ± 3.33 e	25.43 ± 4.29 e	57.56 ± 1.36 b	57.40 ± 7.94 d	3.64 ± 0.49 b	3.59 ± 0.47 b
	2W1S	114.30 ± 13.84 a	111.51 ± 7.96 a	64.06 ± 0.66 a	64.89 ± 2.32 a	5.86 ± 0.63 a	6.07 ± 0.66 a
	2W2S	37.83 ± 1.04 de	36.91 ± 3.27 de	59.53 ± 2.61 ab	59.81 ± 3.55 d	4.78 ± 0.22 ab	4.72 ± 0.49 ab
	3W1S	81.95 ± 11.05 b	76.58 ± 6.68 b	62.72 ± 1.37 ab	63.49 ± 2.97 b	5.84 ± 0.57 a	5.65 ± 0.69 a
	3W2S	67.48 ± 2.59 bc	61.88 ± 3.97 bc	61.47 ± 0.47 ab	62.44 ± 1.98 bc	5.74 ± 0.56 a	5.41 ± 0.72 a
	3W3S	53.62 ± 3.77 cd	48.86 ± 2.51 cd	60.20 ± 2.01 ab	61.85 ± 1.37 cd	5.43 ± 0.73 a	5.16 ± 0.06 ab
Culm	SW	121.54 ± 33.30 d	86.87 ± 10.94 e	53.06 ± 0.52 c	48.37 ± 1.61 b	17.21 ± 4.79 b	12.33 ± 1.16 b
	2W1S	537.16 ± 26.08 a	483.20 ± 29.10 a	63.92 ± 2.11 a	61.97 ± 1.24 a	27.73 ± 1.92 a	26.05 ± 0.86 a
	2W2S	143.91 ± 10.33 d	113.99 ± 14.42 de	54.03 ± 1.99 bc	50.34 ± 2.24 b	18.07 ± 0.11 b	14.65 ± 2.32 b
	3W1S	352.88 ± 19.56 b	310.69 ± 5.82 b	62.64 ± 2.02 a	59.79 ± 1.41 a	25.50 ± 1.99 ab	22.82 ± 1.01 a
	3W2S	263.13 ± 35.44 bc	191.01 ± 2.81 c	60.57 ± 3.10 ab	53.44 ± 1.49 b	22.11 ± 2.70 ab	16.55 ± 1.22 b
	3W3S	191.46 ± 42.26 cd	141.29 ± 20.21 cd	59.26 ± 0.67 abc	51.51 ± 1.73 b	19.45 ± 4.60 ab	14.87 ± 1.67 b
Leaf	SW	166.71 ± 17.96 d	128.52 ± 5.09 e	52.81 ± 5.13 b	46.67 ± 1.22 d	23.55 ± 0.71 c	18.46 ± 1.36 b
	2W1S	567.38 ± 21.78 a	465.03 ± 20.13 a	62.29 ± 2.77 a	61.05 ± 1.29 a	29.23 ± 1.28 a	26.91 ± 1.91 a
	2W2S	197.97 ± 20.35 d	165.20 ± 24.09 de	53.90 ± 4.63 b	49.98 ± 3.67 cd	25.36 ± 1.12 bc	20.86 ± 2.41 ab
	3W1S	380.38 ± 32.66 b	338.00 ± 11.82 b	60.19 ± 2.05 ab	57.84 ± 1.73 ab	27.67 ± 0.64 ab	24.81 ± 1.20 a
	3W2S	299.12 ± 61.50 bc	274.20 ± 36.73 c	57.38 ± 3.32 ab	53.09 ± 2.19 abc	25.72 ± 0.80 abc	23.30 ± 1.38 ab
	3W3S	248.67 ± 30.49 cd	203.51 ± 3.92 d	56.24 ± 3.88 ab	51.40 ± 0.48 bcd	25.44 ± 2.35 abc	21.58 ± 0.76 ab

*Data are expressed as the mean of three replicates ± standard error (n = 3). Values followed by different letters within a column are significantly different (P < 0.05). SW, sole waxy sorghum; 2W1S, two rows of waxy sorghum alternated with one row of soybean; 2W2S, two rows of waxy sorghum alternated with two rows of soybean; 3W1S, three rows of waxy sorghum alternated with one row of soybean; 3W2S, three rows of way sorghum alternated with two rows of soybean; 3W3S, three rows of way sorghum alternated with three rows of soybean*.

### P Accumulation and Transportation of Waxy Sorghum

#### P Content

The P contents of waxy sorghum vegetative organs (including root, culm, and leaf) decreased from the jointing stage to the maturity stage in all treatments (Table 3). Across years and treatments, the P content among organs was in the sequence of leaf > culm > root at the jointing stage, spike > leaf > culm > root at the anthesis stage, grain > leaf > culm > root at the maturity stage. In each growth stage, the P content of each organ under intercropping treatments was higher than that of SW treatment, and the maximum value appeared in the 2W1S treatment. For the mean of 2 years, the P content in 2W1S treatment was higher by 1.93 times for roots, 1.65 times for culms, and 1.65 times for leaves at the jointing stage, 2.17 times for roots, 2.35 times for culms, 2.33 times for leaves, and 2.02 times for spikes at the anthesis stage, and 2.04 times for roots, 2.12 times for culms, 2.03 times for leaves, and 1.73 times for grains at the maturity stage than that of SW treatment.

**Table 3:** Effects of different row ratio configurations on P content (g kg^−1^) of waxy sorghum in the waxy sorghum-soybean intercropping system.

**Organ**	**Treatment**	**2019**			**2020**		
		**Jointing stage**	**Anthesis stage**	**Maturity stage**	**Jointing stage**	**Anthesis stage**	**Maturity stage**
Root	SW	2.03 ± 0.10 e	1.34 ± 0.07 d	0.81 ± 0.04 c	1.98 ± 0.02 e	1.14 ± 0.05 c	1.71 ± 0.04 c
	2W1S	3.92 ± 0.11 a	2.78 ± 0.04 a	1.56 ± 0.03 a	3.85 ± 0.06 a	2.62 ± 0.11 a	1.53 ± 0.12 a
	2W2S	2.29 ± 0.16 de	1.51 ± 0.02 cd	0.92 ± 0.05 c	2.23 ± 0.10 d	1.31 ± 0.17 c	0.75 ± 0.06 c
	3W1S	3.30 ± 0.19 b	2.56 ± 0.11 a	1.46 ± 0.02 a	2.98 ± 0.11 b	2.42 ± 0.10 a	1.45 ± 0.14 a
	3W2S	2.87 ± 0.04 c	2.05 ± 0.13 b	1.17 ± 0.10 b	2.74 ± 0.06 c	1.79 ± 0.10 b	1.09 ± 0.06 b
	3W3S	2.62 ± 0.15 cd	1.62 ± 0.15 c	0.94 ± 0.11 c	2.53 ± 0.03 c	1.42 ± 0.05 c	0.87 ± 0.04 c
Culm	SW	3.30 ± 0.33 d	1.59 ± 0.05 e	1.14 ± 0.16 d	3.26 ± 0.17 c	1.22 ± 0.05 e	0.85 ± 0.15 d
	2W1S	4.95 ± 0.33 a	3.72 ± 0.32 a	2.25 ± 0.12 a	4.78 ± 0.05 a	3.31 ± 0.14 a	2.08 ± 0.04 a
	2W2S	3.49 ± 0.21 cd	1.96 ± 0.12 de	1.33 ± 0.04 d	3.35 ± 0.09 c	2.06 ± 0.08 d	1.35 ± 0.03 c
	3W1S	4.08 ± 0.27 b	3.20 ± 0.15 b	1.99 ± 0.08 ab	3.95 ± 0.08 b	3.07 ± 0.05 b	1.96 ± 0.11 a
	3W2S	3.93 ± 0.34 b	2.76 ± 0.20 c	1.69 ± 0.02 bc	3.87 ± 0.11 b	2.64 ± 0.21 c	1.63 ± 0.08 b
	3W3S	3.58 ± 0.22 c	2.26 ± 0.35 d	1.42 ± 0.11 cd	3.35 ± 0.12 c	2.46 ± 0.14 c	1.57 ± 0.08 b
Leaf	SW	3.65 ± 0.12 d	2.08 ± 0.37 d	1.47 ± 0.05 e	3.62 ± 0.04 d	2.48 ± 0.16 e	1.77 ± 0.28 d
	2W1S	6.07 ± 0.13 a	5.65 ± 0.31 a	3.22 ± 0.04 a	5.98 ± 0.13 a	4.89 ± 0.04 a	3.06 ± 0.15 a
	2W2S	4.24 ± 0.19 d	2.56 ± 0.25 cd	1.65 ± 0.06 e	4.16 ± 0.09 c	2.98 ± 0.05 d	2.17 ± 0.03 cd
	3W1S	5.13 ± 0.30 b	5.01 ± 0.94 a	2.92 ± 0.14 b	4.87 ± 0.29 b	4.52 ± 0.12 b	2.98 ± 0.11 ab
	3W2S	4.85 ± 0.37 bc	3.63 ± 0.43 b	2.26 ± 0.10 c	4.72 ± 0.09 b	3.84 ± 0.10 c	2.55 ± 0.10 bc
	3W3S	4.43 ± 0.07 cd	3.21 ± 0.47 bc	2.04 ± 0.05 d	4.24 ± 0.28 c	3.29 ± 0.15 d	2.29 ± 0.09 c
Spike (Grain)	SW		3.36 ± 0.09 e	4.36 ± 0.07 c		3.23 ± 0.08 d	4.28 ± 0.13 d
	2W1S		5.09 ± 0.13 a	6.47 ± 0.16 a		4.96 ± 0.11 a	6.43 ± 0.11 a
	2W2S		3.76 ± 0.11 d	4.62 ± 0.14 c		3.70 ± 0.14 cd	4.52 ± 0.20 cd
	3W1S		4.52 ± 0.18 b	6.24 ± 0.22 a		4.48 ± 0.44 ab	6.19 ± 0.22 ab
	3W2S		4.23 ± 0.07 c	6.06 ± 0.19 ab		4.16 ± 0.22 bc	5.97 ± 0.21 ab
	3W3S		4.11 ± 0.15 c	5.61 ± 0.10 b		3.99 ± 0.14 bc	5.52 ± 0.19 bc

*Data are expressed as the mean of three replicates ± standard error (n = 3). Different letters within a column and an organ indicate significant differences among treatments (P < 0.05). SW, sole waxy sorghum; 2W1S, two rows of waxy sorghum alternated with one row of soybean; 2W2S, two rows of waxy sorghum alternated with two rows of soybean; 3W1S, three rows of waxy sorghum alternated with one row of soybean; 3W2S, three rows of way sorghum alternated with two rows of soybean; 3W3S, three rows of way sorghum alternated with three rows of soybean*.

#### P Accumulation

**Figure 3:**
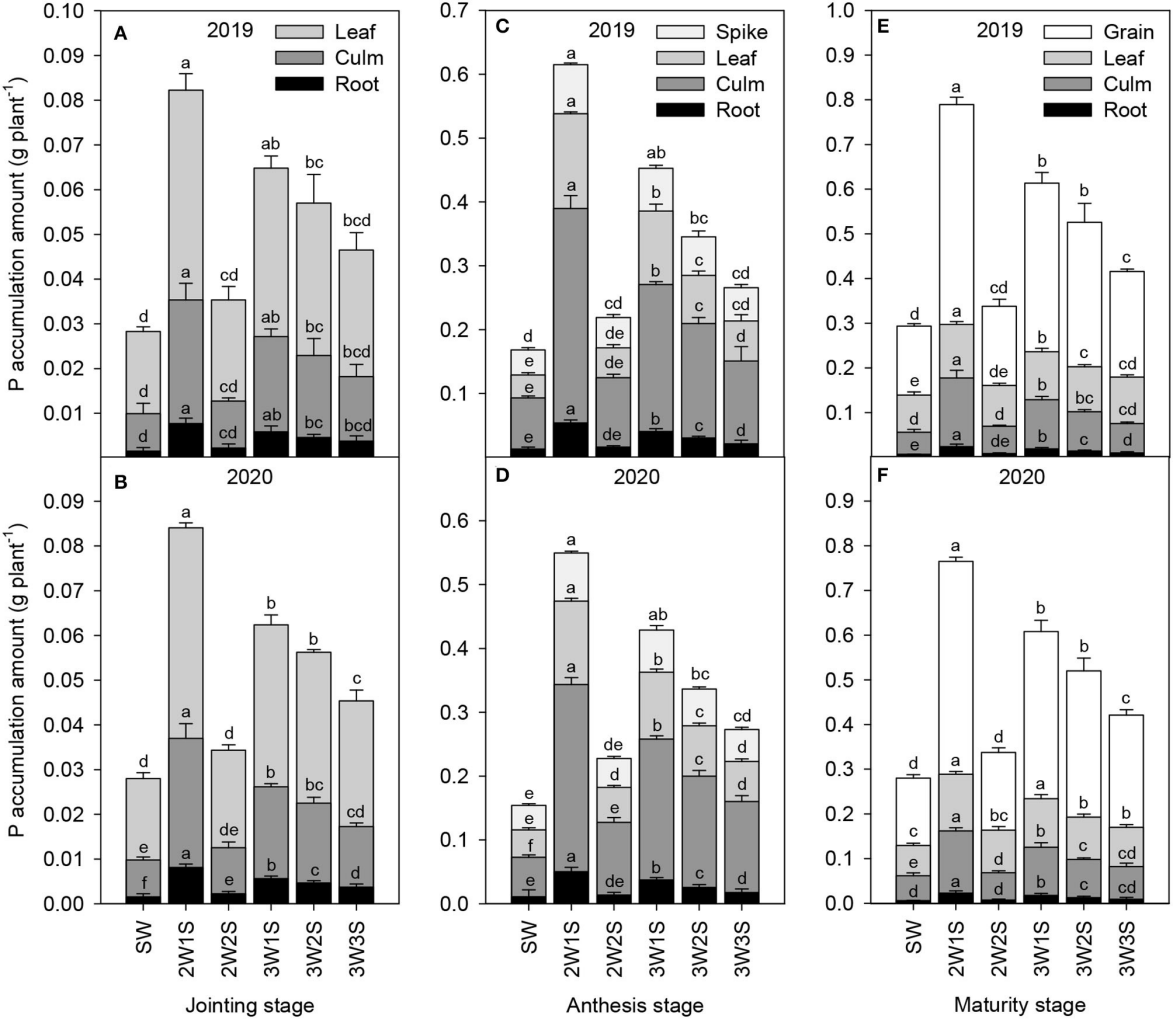
Effects of different row ratio configurations on P accumulation amount of waxy sorghum at the jointing **(A,B)**, anthesis **(C,D)**, and maturity **(E,F)** stage in the waxy sorghum-soybean intercropping system. Data are expressed as the mean of three replicates and bars represent standard errors (*n* = 3). Different letters within a growth stage and an organ indicate significantly differences among treatments (*P* < 0.05). SW, sole waxy sorghum; 2W1S, two rows of waxy sorghum alternated with one row of soybean; 2W2S, two rows of waxy sorghum alternated with two rows of soybean; 3W1S, three rows of waxy sorghum alternated with one row of soybean; 3W2S, three rows of way sorghum alternated with two rows of soybean; 3W3S, three rows of way sorghum alternated with three rows of soybean.

The P accumulation amounts of waxy sorghum vegetative organs (including root, culm, and leaf) increased to their maximum at the anthesis stage and subsequently decreased at the maturity stage in all treatments (Figure 3). In each treatment, the order of P accumulation amount among organs was leaf > culm > root at the jointing stage (Figures 3A,B), spike > leaf > culm > root at the anthesis stage (Figures 3C,D), and grain > leaf > culm > root at the maturity stage (Figures 3E,F). In each growth stage, the P accumulation amount of each organ among treatments was in the sequence of 2W1S > 3W1S > 3W2S > 3W3S > 2W2S > SW, and the 2W1S treatment significantly increased the P accumulation amount (mean of 2 years) by 410.64% for roots, 266.8% for culms, and 195.30% for leaves at the jointing stage, 339.58% for roots, 341.63% for culms, 255.59% for leaves, and 261.36% for spikes at the anthesis stage, and 262.92% for roots, 187.76% for culms, 118.39% for leaves, and 171.02% for grains at the maturity stage compared with the SW treatment.

#### P Transportation

There had significant effects on the PTA, PTR, and GCRPT of waxy sorghum by row ratio configurations in the waxy sorghum-soybean intercropping system (Table 4). Across years and treatments, the PTA, PTR, and GCRPT among organs were in the sequence of culm > leaf > root, root > culm > leaf, and culm > leaf > root, respectively. In both years, the PTA, PTR, and GCRPT of each organ under intercropping treatments were higher than those of SW treatment, and the maximum values appeared in the 2W1S treatment. In particular, compared with the SW treatment, the 2W1S treatment significantly increased the mean PTA, PTR, and GCRPT of 2 years by 428.98, 21.24, and 109.37% for roots, 487.54, 38.3, and 132.72% for culms, and 405.53, 52.08, and 101.93% for leaves, respectively.

**Table 4:** Effects of different row ratio configurations on the P transportation amount before anthesis (PTA), the P transportation rate before anthesis (PTR), and the contribution rate of P transportation before anthesis to grain (GCRPT) of waxy sorghum in the waxy sorghum-soybean intercropping system.

**Organ**	**Treatment**	**PTA (mg plant^−1^)**		**PTR (%)**		**GCRPT (%)**	
		**2019**	**2020**	**2019**	**2020**	**2019**	**2020**
Root	SW	5.98 ± 0.63 e	4.95 ± 0.68 d	46.90 ± 2.03 c	44.78 ± 1.28 c	2.79 ± 0.30 c	2.34 ± 0.25 d
	2W1S	30.14 ± 3.65 a	27.68 ± 1.30 a	55.80 ± 0.97 a	55.35 ± 3.32 a	5.75 ± 0.71 a	5.16 ± 0.19 a
	2W2S	7.59 ± 0.34 de	6.75 ± 1.40 d	47.29 ± 1.06 bc	48.65 ± 2.49 bc	3.18 ± 0.31 c	2.89 ± 0.60 cd
	3W1S	21.77 ± 0.98 b	19.76 ± 0.81 b	53.81 ± 3.01 ab	52.86 ± 1.91 ab	5.11 ± 0.27 ab	4.69 ± 0.35 ab
	3W2S	16.16 ± 1.20 c	13.18 ± 2.57 c	53.48 ± 3.99 b	51.02 ± 2.53 b	4.36 ± 0.67 abc	3.60 ± 0.82 bc
	3W3S	11.21 ± 3.49 cd	8.86 ± 0.82 d	50.35 ± 7.00 b	49.88 ± 5.79 b	3.69 ± 1.18 bc	2.94 ± 0.21 cd
Culm	SW	31.02 ± 7.97 d	26.20 ± 6.74 e	34.85 ± 1.56 c	42.03 ± 2.65 b	14.51 ± 3.78 c	12.31 ± 0.31 c
	2W1S	182.12 ± 27.02 a	154.05 ± 10.92 a	53.84 ± 0.83 a	52.48 ± 2.58 a	33.64 ± 5.16 a	28.78 ± 2.32 a
	2W2S	47.88 ± 7.54 cd	52.52 ± 7.32 d	43.62 ± 4.90 b	45.69 ± 3.71 ab	19.56 ± 1.89 bc	22.31 ± 2.57 b
	3W1S	119.82 ± 9.76 b	112.65 ± 6.40 b	51.98 ± 0.88 a	51.07 ± 1.25 a	27.96 ± 0.86 ab	26.89 ± 0.08 ab
	3W2S	91.30 ± 7.87 bc	88.39 ± 5.76 bc	50.57 ± 0.50 ab	50.68 ± 0.84 a	23.80 ± 0.29 abc	23.45 ± 0.32 ab
	3W3S	64.49 ± 9.24 cd	69.22 ± 6.86 cd	47.67 ± 1.75 ab	48.41 ± 1.01 ab	21.18 ± 6.55 abc	22.94 ± 1.81 b
Leaf	SW	12.97 ± 4.01 d	15.14 ± 5.78 d	31.94 ± 2.37 b	34.85 ± 3.52 b	6.03 ± 0.17 b	7.05 ± 0.79 b
	2W1S	78.67 ± 5.86 a	63.43 ± 0.35 a	52.96 ± 4.14 a	48.61 ± 0.89 a	14.58 ± 1.40 a	11.83 ± 0.14 a
	2W2S	20.01 ± 0.83 cd	19.39 ± 3.44 d	42.75 ± 0.52 ab	35.28 ± 1.44 b	8.34 ± 0.56 b	8.44 ± 1.87 ab
	3W1S	58.06 ± 12.09 b	46.28 ± 6.03 b	49.38 ± 6.51 a	43.92 ± 4.62 ab	13.96 ± 3.48 a	10.90 ± 1.22 a
	3W2S	34.77 ± 1.04 c	34.30 ± 5.76 bc	46.32 ± 1.68 a	42.91 ± 0.81 ab	9.34 ± 1.22 ab	8.96 ± 0.84 ab
	3W3S	28.51 ± 7.88 cd	24.82 ± 2.91 cd	43.85 ± 5.35 a	39.21 ± 2.79 b	9.22 ± 2.39 ab	8.35 ± 1.25 ab

*Data are expressed as the mean of three replicates ± standard error (n = 3). Different letters within a column and an organ indicate significant differences among treatments (P < 0.05). SW, sole waxy sorghum; 2W1S, two rows of waxy sorghum alternated with one row of soybean; 2W2S, two rows of waxy sorghum alternated with two rows of soybean; 3W1S, three rows of waxy sorghum alternated with one row of soybean; 3W2S, three rows of way sorghum alternated with two rows of soybean; 3W3S, three rows of way sorghum alternated with three rows of soybean*.

### K Accumulation and Transportation of Waxy Sorghum

#### K Content

The K contents of waxy sorghum vegetative organs (including root, culm, and leaf) decreased from the jointing stage to the maturity stage in all treatments (Table 5). In both years, the order of N content in each treatment among organs was respectively leaf > culm > root at the jointing stage, spike > leaf > culm > root at the anthesis stage, and grain > leaf > culm > root at the maturity stage. In each growth stage, the K content of each organ among treatments was in the sequence of 2W1S > 3W1S > 3W2S > 3W3S > 2W2S > SW, and the 2W1S treatment significantly increased the mean K content of 2 years by 20.58% for roots, 41.03% for culms, and 46.51% for leaves at the jointing stage, 28.78% for roots, 61.37% for culms. 68.15% for leaves, 63.16% for spikes at the anthesis stage, 21.10% for roots, 52.09% for culms, 55.09% for leaves, and 52.64% for grains at the maturity stage compared with the SW treatment.

**Table 5:** Effects of different row ratio configurations on K content (g kg^−1^) of waxy sorghum in the waxy sorghum-soybean intercropping system.

**Organ**	**Treatment**	**2019**			**2020**		
		**Jointing stage**	**Anthesis stage**	**Maturity stage**	**Jointing stage**	**Anthesis stage**	**Maturity stage**
Root	SW	21.90 ± 0.63 c	15.66 ± 0.52 d	12.17 ± 0.71 c	21.20 ± 1.11 e	15.55 ± 1.87 c	11.69 ± 1.02 c
	2W1S	26.11 ± 0.68 a	20.50 ± 3.81 a	14.48 ± 0.41 a	25.85 ± 1.20 a	19.70 ± 1.65 a	14.42 ± 0.60 a
	2W2S	22.46 ± 0.19 c	16.87 ± 1.26 cd	12.77 ± 0.80 bc	22.21 ± 0.32 de	17.54 ± 1.66 b	12.56 ± 0.08 bc
	3W1S	25.80 ± 0.65 a	19.77 ± 3.02 ab	14.33 ± 0.51 a	25.01 ± 0.98 ab	19.30 ± 2.03 a	14.16 ± 0.67 a
	3W2S	24.38 ± 0.77 b	18.82 ± 2.63 abc	14.21 ± 0.30 a	24.20 ± 0.79 bc	18.52 ± 0.42 ab	13.38 ± 1.23 ab
	3W3S	23.85 ± 0.18 b	17.31 ± 1.11 bcd	13.50 ± 0.63 ab	23.53 ± 0.71 cd	17.60 ± 0.75 b	13.17 ± 0.68 ab
Culm	SW	20.69 ± 0.60 e	13.88 ± 0.45 d	12.17 ± 0.58 d	20.48 ± 0.78 e	12.26 ± 1.45 e	10.96 ± 0.33 e
	2W1S	33.66 ± 0.71 a	26.31 ± 0.54 a	21.41 ± 1.09 a	33.20 ± 1.04 a	26.04 ± 1.77 a	21.16 ± 0.75 a
	2W2S	22.62 ± 1.07 de	16.34 ± 0.60 cd	14.21 ± 0.55 cd	22.51 ± 2.76 de	15.97 ± 1.78 d	13.95 ± 0.74 d
	3W1S	30.70 ± 2.60 ab	23.25 ± 1.77 ab	19.10 ± 0.80 ab	29.35 ± 2.52 b	23.10 ± 2.79 ab	18.73 ± 1.00 b
	3W2S	26.79 ± 0.64 bc	20.48 ± 1.66 b	17.38 ± 0.71 bc	26.41 ± 1.13 c	19.83 ± 1.66 bc	16.97 ± 0.36 bc
	3W3S	23.73 ± 3.71 cd	19.64 ± 0.25 bc	16.82 ± 1.78 bc	23.13 ± 1.68 d	18.47 ± 1.08 cd	16.24 ± 0.56 c
Leaf	SW	18.91 ± 0.57 c	11.60 ± 0.89 e	9.68 ± 0.82 d	18.66 ± 1.66 c	11.44 ± 0.51 d	9.51 ± 0.15 d
	2W1S	30.11 ± 1.33 a	21.50 ± 1.09 a	15.68 ± 0.79 a	29.54 ± 0.63 a	21.13 ± 1.33 a	15.49 ± 0.50 a
	2W2S	25.78 ± 0.74 bc	14.68 ± 0.45 d	12.09 ± 0.92 c	25.65 ± 0.42 b	12.97 ± 0.14 d	10.74 ± 0.37 cd
	3W1S	28.72 ± 1.73 a	19.34 ± 1.42 ab	14.58 ± 1.94 ab	28.33 ± 1.17 ab	18.16 ± 0.37 b	14.12 ± 2.02 ab
	3W2S	27.36 ± 2.92 ab	17.37 ± 1.74 bc	13.26 ± 2.39 bc	27.11 ± 0.79 ab	16.82 ± 0.30 bc	13.08 ± 0.52 abc
	3W3S	26.76 ± 1.00 abc	16.05 ± 1.40 cd	12.46 ± 2.24 bc	26.06 ± 1.92 b	15.40 ± 0.22 c	12.41 ± 0.33 bc
Spike (Grain)	SW		8.94 ± 0.21 d	10.21 ± 0.34 e		8.78 ± 0.95 b	10.14 ± 0.32 c
	2W1S		12.52 ± 0.82 a	15.45 ± 0.18 a		12.38 ± 0.47 a	13.99 ± 0.38 a
	2W2S		9.52 ± 0.81 cd	11.58 ± 1.50 de		9.47 ± 0.18 b	11.25 ± 0.72 bc
	3W1S		11.24 ± 0.63 b	13.83 ± 0.17 b		10.92 ± 0.96 ab	13.69 ± 0.67 a
	3W2S		10.46 ± 0.65 bc	13.29 ± 0.66 bc		10.29 ± 1.84 ab	13.65 ± 1.18 a
	3W3S		10.11 ± 0.33 c	12.28 ± 0.52 cd		9.71 ± 0.49 b	12.07 ± 0.14 b

*Data are expressed as the mean of three replicates ± standard error (n = 3). Different letters within a column and an organ indicate significant differences among treatments (P < 0.05). SW, sole waxy sorghum; 2W1S, two rows of waxy sorghum alternated with one row of soybean; 2W2S, two rows of waxy sorghum alternated with two rows of soybean; 3W1S, three rows of waxy sorghum alternated with one row of soybean; 3W2S, three rows of way sorghum alternated with two rows of soybean; 3W3S, three rows of way sorghum alternated with three rows of soybean*.

#### K Accumulation

**Figure 4:**
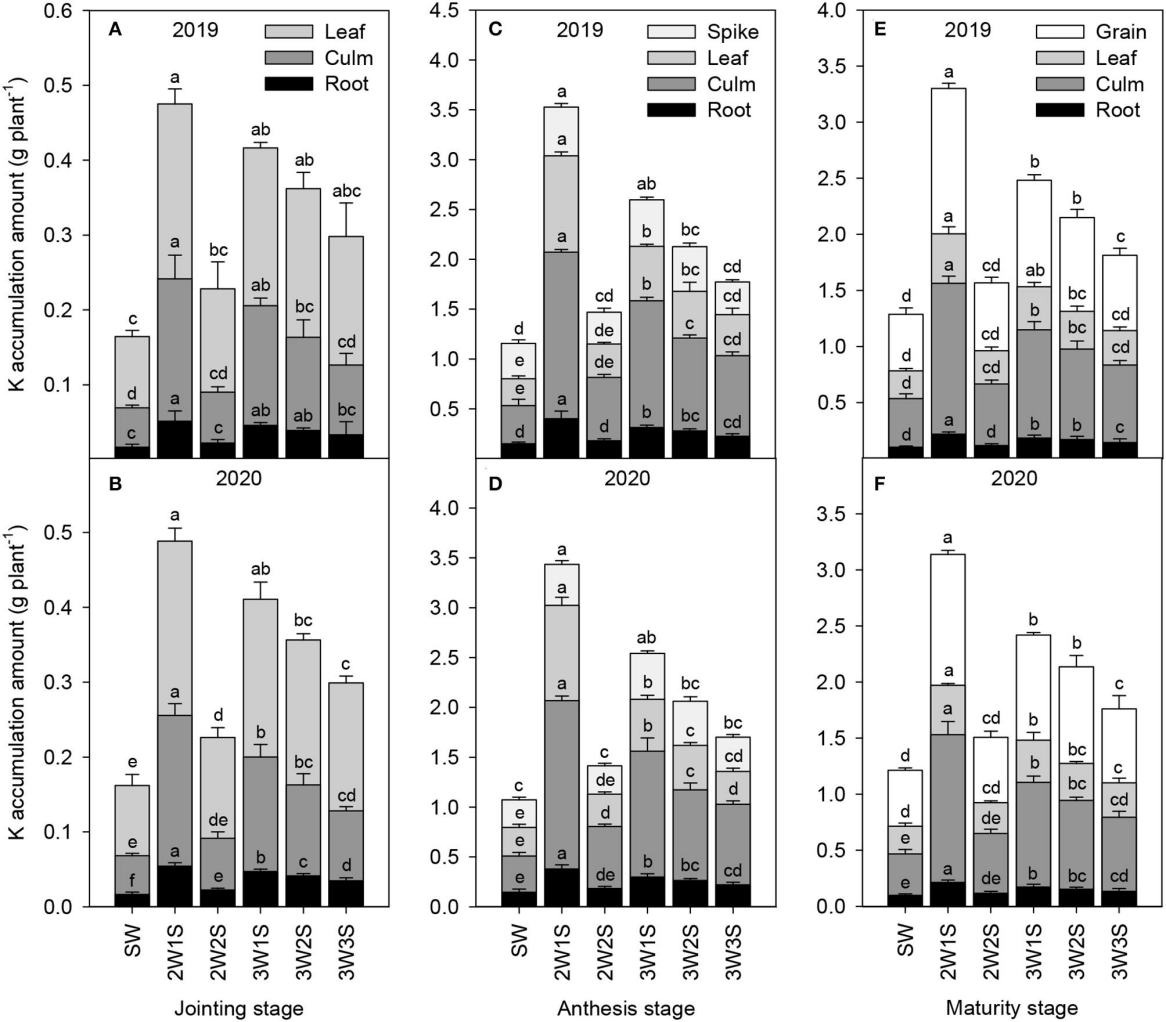
Effects of different row ratio configurations on K accumulation amount of waxy sorghum at the jointing **(A,B)**, anthesis **(C,D)**, and maturity **(E,F)** stage in the waxy sorghum-soybean intercropping system. Data are expressed as the mean of three replicates and bars represent standard errors (*n* = 3). Different letters within a growth stage and an organ indicate significantly differences among treatments (*P* < 0.05). SW, sole waxy sorghum; 2W1S, two rows of waxy sorghum alternated with one row of soybean; 2W2S, two rows of waxy sorghum alternated with two rows of soybean; 3W1S, three rows of waxy sorghum alternated with one row of soybean; 3W2S, three rows of way sorghum alternated with two rows of soybean; 3W3S, three rows of way sorghum alternated with three rows of soybean.

The K accumulation amounts of waxy sorghum vegetative organs (including root, culm, and leaf) increased from the jointing stage to the anthesis stage but decreased from the anthesis stage to the maturity stage in all treatments (Figure 4). Across years and treatments, the order of K accumulation amount among organs was leaf > culm > root at the jointing stage (Figures 4A,B), spike > leaf > culm > root at the anthesis stage (Figures 4C,D), and grain > leaf > culm > root at the maturity stage (Figures 4E,F). In each growth stage, the K accumulation amount of each organ under intercropping treatments was higher than that of SW treatment, and the maximum value appeared in the 2W1S treatment. In particular, the K accumulation amount (mean of 2 years) in the 2W1S treatment was higher by 3.19 times for roots, 3.61 times for culms, and 2.95 times for leaves at the jointing stage, 2.63 times for roots, 3.97 times for culms, 3.79 times for leaves, and 3.12 times for spikes at the anthesis stage, and 2.16 times for roots, 3.15 times for culms, 2.65 times for leaves, and 2.58 times for grains at the maturity stage than that of SW treatment, respectively.

#### K Transportation

The row ratio configurations had significant effects on the KTA, KTR, and GCRKT of waxy sorghum in the waxy sorghum-soybean intercropping system (Table 6). In each treatment, the KTA, KTR, and GCRKT among organs were in the sequence of culm > leaf > root, root > leaf > culm, and culm > leaf > root, respectively. In both years, the KTA, KTR, and GCRKT of each organ among treatments were in the sequence of 2W1S > 3W1S > 3W2S > 3W3S > 2W2S > SW. In particular, the KTA, KTR, and GCRKT (mean of 2 years) in the 2W1S treatment were higher, respectively, by 3.61, 1.34, and 1.44 times for roots, 5.57, 1.64, and 2.26 times for culms, and 4.50, 1.57, and 1.84 times for leaves than that of SW treatment.

**Table 6:** Effects of different row ratio configurations on the K transportation amount before anthesis (KTA), the K transportation rate before anthesis (KTR), and the contribution rate of K transportation before anthesis to grain (GCRKT) of waxy sorghum in the waxy sorghum-soybean intercropping system.

**Organ**	**Treatment**	**KTA (mg plant^−1^)**		**KTR (%)**		**GCRKT (%)**	
		**2019**	**2020**	**2019**	**2020**	**2019**	**2020**
Root	SW	47.68 ± 5.90 d	49.19 ± 4.41 d	31.69 ± 5.58 d	33.44 ± 0.50 b	9.52 ± 0.18 c	9.93 ± 0.19 b
	2W1S	183.99 ± 15.11 a	166.08 ± 7.70 a	43.17 ± 2.67 a	44.06 ± 0.51 a	14.04 ± 1.85 a	13.92 ± 0.52 a
	2W2S	61.79 ± 3.27 d	67.07 ± 5.10 cd	34.56 ± 2.74 cd	37.85 ± 2.85 ab	10.35 ± 0.79 bc	10.74 ± 0.41 ab
	3W1S	129.23 ± 15.69 b	125.94 ± 14.05 ab	41.46 ± 0.66 ab	41.91 ± 1.40 ab	13.80 ± 0.22 ab	13.49 ± 0.55 a
	3W2S	106.88 ± 20.10 bc	111.51 ± 10.43 bc	37.78 ± 0.45 abc	41.76 ± 6.14 ab	13.35 ± 1.08 ab	13.34 ± 1.48 a
	3W3S	81.56 ± 6.06 cd	57.41 ± 4.49 bcd	35.96 ± 3.38 bcd	39.06 ± 3.58 ab	11.53 ± 1.22 abc	11.54 ± 0.04 a
Culm	SW	170.64 ± 17.12 c	153.98 ± 34.89 e	23.23 ± 2.29 c	23.83 ± 2.02 c	34.85 ± 5.41 c	30.33 ± 1.09 d
	2W1S	924.23 ± 41.10 a	884.17 ± 24.43 a	39.00 ± 0.20 a	38.39 ± 1.12 a	71.51 ± 2.30 a	75.52 ± 1.27 a
	2W2S	252.89 ± 32.80 c	243.40 ± 23.80 de	27.94 ± 1.25 b	25.16 ± 3.00 bc	41.90 ± 0.99 bc	41.69 ± 3.22 c
	3W1S	606.54 ± 25.34 ab	629.67 ± 12.94 b	36.27 ± 2.24 a	35.93 ± 3.46 ab	63.67 ± 1.33 a	66.95 ± 1.38 b
	3W2S	427.62 ± 32.68 bc	417.89 ± 20.91 c	30.43 ± 4.08 b	31.62 ± 3.24 abc	48.81 ± 3.41 b	48.16 ± 1.55 c
	3W3S	317.45 ± 39.88 bc	302.49 ± 36.24 d	28.23 ± 3.55 b	28.01 ± 3.00 bc	47.77 ± 1.90 b	45.77 ± 2.73 c
Leaf	SW	49.98 ± 10.26 d	50.34 ± 11.26 c	25.25 ± 1.06 e	25.06 ± 1.37 c	9.87 ± 0.18 c	10.14 ± 0.89 b
	2W1S	224.66 ± 14.94 a	226.30 ± 25.85 a	39.72 ± 0.43 a	39.22 ± 1.74 a	17.32 ± 0.86 a	19.52 ± 1.91 a
	2W2S	72.74 ± 7.19 cd	70.55 ± 8.72 bc	28.14 ± 2.07 de	26.52 ± 0.53 c	12.24 ± 1.07 bc	11.08 ± 0.95 b
	3W1S	160.46 ± 11.18 ab	143.93 ± 29.61 ab	36.70 ± 2.86 ab	36.49 ± 1.20 b	17.06 ± 1.15 ab	15.28 ± 1.49 ab
	3W2S	130.78 ± 4.84 bc	116.37 ± 5.88 bc	34.80 ± 3.79 bc	33.65 ± 0.13 b	15.82 ± 1.93 ab	13.54 ± 0.31 ab
	3W3S	103.92 ± 9.11 bcd	87.44 ± 19.57 bc	31.00 ± 3.88 cd	28.07 ± 1.22 c	15.32 ± 2.44 ab	12.03 ± 3.89 b

*Data are expressed as the mean of three replicates ± standard error (n = 3). Different letters within a column and an organ indicate significant differences among treatments (P < 0.05). SW, sole waxy sorghum; 2W1S, two rows of waxy sorghum alternated with one row of soybean; 2W2S, two rows of waxy sorghum alternated with two rows of soybean; 3W1S, three rows of waxy sorghum alternated with one row of soybean; 3W2S, three rows of way sorghum alternated with two rows of soybean; 3W3S, three rows of way sorghum alternated with three rows of soybean*.

### Yield and Economic Benefits

**Figure 5:**
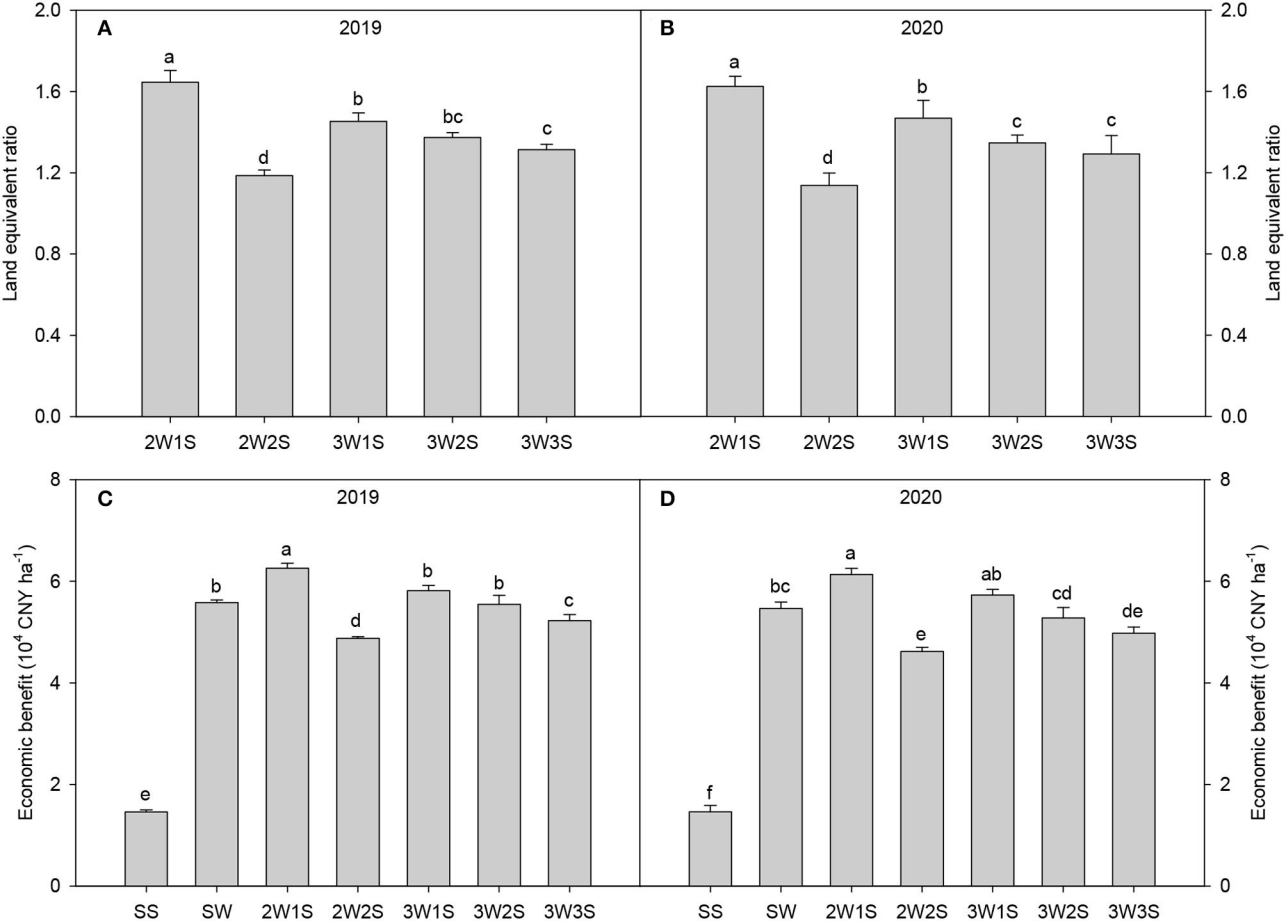
Effects of different row ratio configurations on the land equivalent ratio **(A,B)** and economic benefits **(C,D)** in the waxy sorghum-soybean intercropping system. Data are expressed as the mean of three replicates and bars represent standard errors (*n* = 3). Different letters indicate significantly differences among treatments (*P* < 0.05). SS, sole soybean; SW, sole waxy sorghum; 2W1S, two rows of waxy sorghum alternated with one row of soybean; 2W2S, two rows of waxy sorghum alternated with two rows of soybean; 3W1S, three rows of waxy sorghum alternated with one row of soybean; 3W2S, three rows of way sorghum alternated with two rows of soybean; 3W3S, three rows of way sorghum alternated with three rows of soybean.

There had significant effects on the yield and yield components of waxy sorghum by row ratio configurations in the waxy sorghum-soybean system (Table 7). In both years, intercropping increased the spike length, the grain number per spike, and the 1,000-grain weight of waxy sorghum while decreasing the yields of waxy sorghum and soybean. The spike length, the grain number per spike, and the 1,000-grain weight of waxy sorghum among treatments were in the sequence of 2W1S > 3W1S > 3W2S > 3W3S > 2W2S > SW, while the yields of waxy sorghum and soybean among treatments were in the sequence of SW (SS) > 2W1S > 3W2S > 3W1S > 2W2S > 3W3S. Besides, the maximum land equivalent ratio (Figures 5A,B) and economic benefit (Figures 5C,D) appeared in the 2W1S treatment, and the mean of 2 years was 1.64 and 5.52 × 10^4^ CNY ha^−1^, respectively.

**Table 7:** Effects of different row ratio configurations on yields of waxy sorghum and soybean in the waxy sorghum-soybean intercropping system.

**Year**	**Treatment**	**Yield components of waxy sorghum**			**Yield (kg ha^−1^)**	
		**Spike length (cm)**	**Grain number per spike**	**1,000-grain weight (g)**	**Waxy sorghum**	**Soybean**
2019	SW (SS)	31.75 ± 0.57 b	2845.95 ± 36.24 b	17.80 ± 0.36 b	5582.61 ± 49.25 a	2430.18 ± 69.70 a
	2W1S	33.91 ± 0.23 a	3769.96 ± 68.34 a	20.83 ± 0.69 a	5225.85 ± 79.31 b	1718.71 ± 57.73 b
	2W2S	32.31 ± 0.25 ab	3037.19 ± 254.71 b	18.31 ± 1.09 ab	4263.68 ± 53.55 e	1023.37 ± 66.45 d
	3W1S	33.43 ± 0.10 ab	3466.85 ± 161.55 a	20.77 ± 1.43 a	5006.54 ± 110.65 bc	1348.05 ± 23.98 c
	3W2S	32.70 ± 2.46 ab	3108.82 ± 46.00 b	18.75 ± 1.04 ab	4794.50 ± 155.53 cd	1254.22 ± 54.23 c
	3W3S	32.38 ± 0.43 ab	3066.61 ± 94.92 b	18.40 ± 0.42 ab	4479.16 ± 104.60 de	1241.73 ± 47.57 c
2020	SW (SS)	31.06 ± 0.19 d	2804.79 ± 52.22 d	18.02 ± 0.32 d	5465.79 ± 124.66 a	2437.33 ± 211.05 a
	2W1S	33.88 ± 0.20 a	3771.21 ± 70.13 a	21.04 ± 0.20 a	5149.75 ± 112.48 ab	1641.85 ± 21.92 b
	2W2S	32.05 ± 0.08 cd	3003.59 ± 24.70 cd	18.42 ± 0.56 cd	4048.28 ± 81.14 e	949.68 ± 9.36 d
	3W1S	33.44 ± 0.35 ab	3414.29 ± 186.91 b	20.95 ± 0.10 a	4913.62 ± 106.43 bc	1361.24 ± 10.02 bc
	3W2S	32.41 ± 0.15 bc	3106.24 ± 75.15 c	19.34 ± 0.61 b	4531.64 ± 227.95 cd	1243.76 ± 39.64 cd
	3W3S	32.18 ± 1.47 cd	3042.56 ± 44.38 cd	18.76 ± 0.23 bc	4241.03 ± 106.83 cd	1228.39 ± 40.40 cd

*Data are expressed as the mean of three replicates ± standard error (n = 3). Different letters within a column and an organ indicate significant differences among treatments (P < 0.05). SW, sole waxy sorghum; 2W1S, two rows of waxy sorghum alternated with one row of soybean; 2W2S, two rows of waxy sorghum alternated with two rows of soybean; 3W1S, three rows of waxy sorghum alternated with one row of soybean; 3W2S, three rows of way sorghum alternated with two rows of soybean; 3W3S, three rows of way sorghum alternated with three rows of soybean*.

## Discussion

Nitrogen uptake and utilization by crops is a key process of the N cycle in agricultural ecosystems and an important basis for crop yield formation (Wu et al., 2015a). Intercropping, especially gramineas and legume intercropping systems play an important role in improving N uptake and utilization of gramineas crops (Yang et al., 2015; Liang et al., 2020). For instance, Gooding et al. (2007) reported that wheat-faba bean and wheat-pea intercropping systems increased the N concentration of wheat grain. Chen et al. (2017) found that the maize-soybean intercropping system improved N concentrations and accumulation amounts of straws and grains in maize. In the present study, the N contents and accumulation amounts of each organ under intercropping treatments were higher than those of SW treatment, suggesting that the waxy sorghum-soybean intercropping system can increase the N absorption among organs in waxy sorghum. These increments in N contents and accumulation amounts may be attributed to the following two possibilities. On the one hand, this might be attributed to the waxy sorghum-soybean intercropping system improving the light environment of the waxy sorghum canopy and promoting the N synthetic of each organ in waxy sorghum (Wang et al., 2021a). On the other hand, another possible explanation might be related to the transfer of N fixation from soybean to waxy sorghum, which is consistent with a previous study on the oat-mung bean intercropping system (Yang et al., 2015). Our results showed that the N accumulation amounts of waxy sorghum vegetative organs (including root, culm, and leaf) increased from the jointing stage to the anthesis stage but decreased from the anthesis stage to the maturity stage in all treatments, indicating that the key period of N accumulation in waxy sorghum is from the anthesis to maturity stages, and the similar result was showed in proso millet under the proso millet-mung bean intercropping system (Dang et al., 2019). Furthermore, we also observed that the NTA, the NTR, and the GCRNT of waxy sorghum among treatments were in the sequence of 2W1S > 3W1S > 3W2S > 3W3S > 2W2S > SW, implying that the waxy sorghum-soybean intercropping system can promote the N transportation from vegetative organs to grains in waxy sorghum, which is an agreement with the previous finding on wheat-peanut (Liu et al., 2019) and maize-pea (Zhao et al., 2019) intercropping systems. These results indicate that the waxy sorghum-soybean intercropping system can increase the N absorption among organs and promote the N transportation from vegetative organs to grains in waxy sorghum.

In this study, the P accumulation amounts of waxy sorghum vegetative organs (including root, culm, and leaf) increased to their maximum at the anthesis stage and subsequently decreased at the maturity stage in all treatments. This result is similar to our previous study on dry matter accumulation (Wang et al., 2021a), and suggests that the key period of P accumulation in waxy sorghum is from the anthesis stage to the maturity stage. Additionally, we found that the N contents and accumulation amounts of roots, culms, leaves, spikes, and grains in waxy sorghum under intercropping treatments were higher as compared to those organs under SW treatment, indicating that the waxy sorghum-soybean intercropping system can increase the P absorption among organs in waxy sorghum. Similar to our results, Ndayisaba et al. (2021) reported an increase of available P in maize under the maize-desmodium intercropping system than in the sole cropping system. Likely, there were three possible processes that might have influenced the P absorption in waxy sorghum as its content and accumulation amount increased under the waxy sorghum-soybean intercropping system. The first was soybean roots could secrete carboxylates, protons, phosphatase, and phytase to increase the P availability in the rhizosphere of waxy sorghum, and thus promoted the P acquisition of waxy sorghum plants (Zhou et al., 2019). The first was soybean roots could secrete carboxylates, protons, phosphatase, and phytase to increase the P availability in the rhizosphere of waxy sorghum, and thus promoted the P acquisition of waxy sorghum plants (Wang et al., 2020b; Bai et al., 2021). The third was reduced number of waxy sorghum plants under intercropping treatments that would increase the P reallocation, improving P content and accumulation amount of different organs in waxy sorghum (Raza et al., 2019). In the present study, the PTA, the PTR, and the GCRPT of each organ under intercropping treatments were higher than those of SW treatment in both years, suggesting that the waxy sorghum-soybean intercropping system can promote the P transportation from vegetative organs to grains in waxy sorghum. Consequently, the waxy sorghum-soybean intercropping system can increase the P absorption among organs and promote the P transportation from vegetative organs to grains in waxy sorghum.

At present, there are few reports on K uptake, accumulation, and transportation of crops in intercropping systems. In this study, we measured the differences in K uptake, accumulation, and transportation of different organs in waxy sorghum under the waxy sorghum-soybean system. Similar to N and P, the K contents and accumulation amounts of each organ under intercropping treatments were higher than those of SW treatment. These increments in K contents and accumulation amounts might be due to the intercropping system promoting K absorption by the charge compensation mechanism while promoting the N absorption of waxy sorghum (Han et al., 2021; Yin et al., 2021). The results of the present study indicate that the waxy sorghum-soybean intercropping system can increase the K absorption among organs in waxy sorghum. Contrary to our findings, Raza et al. (2019) reported that lower K uptake in intercropped maize has appeared in the maize-soybean intercropping system. This inconsistency requires further study. In the same way, we observed that the KTA, the KTR, and the GCRKT of each organ under intercropping treatments were higher than those of SW treatment in both years, suggesting that the waxy sorghum-soybean intercropping system can promote the K transportation from vegetative organs to grain in waxy sorghum. It is worth mentioning that the GCRNT, GCRPT, and GCRKT among organs in waxy sorghum were in the sequence of leaf > culm > root, culm > leaf > root, and culm > leaf > root, respectively. These results indicate that the leaf is the most important N supply source for waxy sorghum grain and the culm is the most important P and K supply source for waxy sorghum grain.

In our study, the waxy sorghum-soybean intercropping system decreased the yield of waxy sorghum; this is because the waxy sorghum in intercropping treatments had a lower number of effective spikes as compared to SW treatment. Similar, intercropping treatments reduced the soybean yield as compared to SS treatment; this may be due to the shading of waxy sorghum to soybean and a decrease in the effective plants' number of soybean (Wu et al., 2015b; Li et al., 2019a). However, the waxy sorghum-soybean intercropping system increased the spike length, the grain number per spike, and the 1,000-grain weight of waxy sorghum, implying that the waxy sorghum-soybean intercropping system can promote the growth and development of spikes in waxy sorghum. Row ratio configuration is an important agronomic measure in intercropping systems, and a reasonable row ratio configuration can construct a suitable field microclimate environment to promote the effective utilization of resources by crops (Okonji et al., 2020; Wang et al., 2020a). In this study, the 2W1S treatment presented the lowest yield, decreasing the amplitude and highest N content and accumulation amount, P content and accumulation amount, K content and accumulation amount, NTA, NTR, GCRNT, PTA, PTR, GCRPT, KTA, KTR, GCRKT, and yield components of waxy sorghum. These changes may be due to the following three reasons. The first was that the 2W1S treatment created a more suitable light environment, increased the photosynthetic active radiation and the leaf area index of the waxy sorghum canopy, and improved the photosynthetic performance of sorghum leaves (Wang et al., 2021a). The second was that the proportion of waxy sorghum plants was more suitable, and its competitiveness was higher than that of soybean, which was conducive to giving full play to the resource utilization advantage of waxy sorghum (Feng et al., 2022). The third was that the 2W1S treatment could give full play to the nitrogen fixation effect of soybean and promote the nutrient transfer from soybean to waxy sorghum (Raza et al., 2019). Overall, the 2W1S treatment showed the highest land equivalent ratio and economic benefit so as to suggest that the 2W1S was the optimal row ratio configuration of the waxy sorghum-soybean system.

## Conclusion

Row ratio configurations had significant effects on nutrient (N, P, and K) accumulation and the transportation of waxy sorghum in the waxy sorghum-soybean intercropping system. The waxy sorghum-soybean intercropping system can increase the N, P, and K absorption among organs and promote the N, P, and K transportation from vegetative organs to grains in waxy sorghum so as to promote the growth and development of spikes in waxy sorghum to obtain higher land equivalent ratio and economic benefits. The 2W1S should be used as the optimal row ratio configuration of the waxy sorghum-soybean system to attain the maximum utilization of nutrient resources.

## Data Availability Statement

The raw data supporting the conclusions of this article will be made available by the authors, without undue reservation.

## Author Contributions

CW and MS conceived and designed the experiments. CW analyzed the data and wrote the manuscript. CW, LZ, and JG carried out the field experiments. GZ, FP, CZ, QZ, and QP sampled the plants and collected the data. MS revised the manuscript. All authors contributed to the manuscript enhancement and finalization and approved the final version of the manuscript.

## Funding

This work was supported by the China Agriculture Research System of MOF and MARA (CARS-06-14.5-B26), the Science and Technology Program of Guizhou Province (QKHZC20201Y053 and QKHZC20201Y122), the Special Funds for Industrial Technology System Construction of Characteristic Multigrain in Guizhou (QCN201881), and the Technological Innovation Project of Kweichow Moutai Co., LTD (2021002).

## Conflict of Interest

The authors declare that the research was conducted in the absence of any commercial or financial relationships that could be construed as a potential conflict of interest. The handling editor declared a shared affiliation with the authors at the time of the review.

## Publisher's Note

All claims expressed in this article are solely those of the authors and do not necessarily represent those of their affiliated organizations, or those of the publisher, the editors and the reviewers. Any product that may be evaluated in this article, or claim that may be made by its manufacturer, is not guaranteed or endorsed by the publisher.
